# Differences in Fat-Free Mass According to Serum Vitamin D Level and Calcium Intake: Korea National Health and Nutrition Examination Survey 2008–2011

**DOI:** 10.3390/jcm10225428

**Published:** 2021-11-20

**Authors:** Hye-Ji An, Young-Gyun Seo

**Affiliations:** Department of Family Medicine, Hallym University Sacred Heart Hospital, Anyang-si 14068, Gyeonggi-do, Korea; hjian90@hallym.or.kr

**Keywords:** body composition, fat-free mass, muscle, sarcopenia, vitamin D, calcium

## Abstract

We analyzed the differences in fat-free mass (FFM) according to serum vitamin D level (VitD) and daily calcium intake (Ca) in 14,444 adults aged over 19 years. We used data from the 4th and 5th Korea National Health and Nutrition Examination Surveys (2008–2011). FFM was measured using dual-energy X-ray absorptiometry. VitD was classified as insufficient or sufficient (cutoff: 20 ng/mL). Ca was classified as unsatisfactory or satisfactory (recommended daily intake: 700 mg). In men, the FFM of group 2 (VitD ≥ 20 ng/mL; Ca < 700 mg), group 3 (VitD < 20 ng/mL; Ca ≥ 700 mg) and group 4 (VitD ≥ 20 ng/mL; Ca ≥ 700 mg) was 0.50 kg (95% confidence interval (CI), 0.084–0.92), 0.78 kg (95% CI, 0.26–1.29) and 1.58 kg (95% CI, 0.95–2.21) higher than that of group 1 (VitD < 20 ng/mL; Ca < 700 mg), respectively. In women, a 1 ng/mL increase in VitD was associated with a 0.023 kg increase in FFM (95% CI, 0.003–0.043) and a 1 g increase in Ca was associated with a 0.62 kg increase in FFM (95% CI, 0.067–1.16). High VitD and Ca were associated with a high FFM.

## 1. Introduction

The prevalence of vitamin D deficiency in Korea in 2014 was 75.2% in men and 82.5% in women [1] and the average daily calcium intake of individuals aged over 50 years was only 470 mg/day in 2008–2010 [2].

Vitamin D and calcium levels are known to be related to body muscle mass and bone mass. There is ample evidence on the association of serum vitamin D levels with overweight, obesity [3,4], body fat mass [5] and regulation of adipogenesis and fat metabolism [6]. Furthermore, low serum vitamin D levels have been found to increase the risk of muscle weakness and sarcopenia [7].

Likewise, many studies have found associations of calcium intake with body weight [8], body adiposity [9] and body composition [10,11], possibly because calcium plays a significant role in the regulation of lipogenesis, lipolysis and energy metabolism [12]. In a 10-year longitudinal study, low serum calcium levels were found to reflect significant muscle loss in adults aged over 50 years and low calcium intake was significantly associated with muscle loss in women [13].

Muscle mass is an important source of energy expenditure. A previous study found that skeletal muscle metabolism is a major determinant of resting energy expenditure [14]. Therefore, factors that increase muscle mass can even lead to a decrease in body fat mass. In a randomized controlled trial of the combined effect of vitamin D and calcium, it was found that calcium and vitamin D intake promoted visceral fat loss in individuals with a very low calcium intake [15]. Moreover, it has been reported that calcium and vitamin D supplementation improves muscle function [16].

The prevention of sarcopenia is crucial because it can impair physical capability, increase the risk of falls and lead to dependence [17]. By elucidating the relationship among vitamin D, calcium and muscle mass, it may be possible to prevent sarcopenia through the improvement of nutrition intake. However, few studies have simultaneously considered vitamin D and calcium and analyzed their relationship with body composition. Therefore, we grouped the Korean general population based on their serum vitamin D level and daily calcium intake and analyzed the differences in body composition, especially fat-free mass (FFM), among them.

## 2. Materials and Methods

### 2.1. Study Design and Participants

The Korea Disease Control and Prevention Agency has conducted the Korea National Health and Nutrition Examination Surveys (KNHANES) since 1998 to comprehensively examine the health, nutritional and socioeconomic status of Korean individuals. We screened 28,377 participants aged over 19 years whose data were collected in the fourth and fifth survey (2008–2011). Among them, 13,933 were excluded because of decreased renal function (estimated glomerular filtration rate < 30), history of diagnosed cancer, inappropriate fasting duration before sample collection (>24 h or <8 h), inappropriate nutritional intake (<500 or >5000 kcal/day), excessive water intake per kilogram body weight (≥90 g/kg) and missing survey records or test results. Consequently, data from 14,444 participants (5856 men and 8588 women) were used in this study.

All procedures were approved by the Ethics Committee of the Korea Disease Control and Prevention Agency (approval numbers 2011-02CON-06-C, 2010-02CON-21-C, 2009-01CON-03-2C and 2008-04EXP-01-C) and were carried out in accordance with the ethical standards laid down in the 1964 Declaration of Helsinki and its later amendments. Signed informed consent was obtained from all KNHANES participants. The KNHANES data are publicly available.

### 2.2. Measurements

Blood samples and body composition data were collected on the same day. Body composition was measured using dual energy X-ray absorptiometry (Hologic Discovery, Hologic, Marlborough, MA, USA). It included measurement of the whole-body total FFM (including bone mineral content (BMC)) and whole-body total BMC. FFM was defined as FFM data obtained from dual energy X-ray absorptiometry minus BMC.

We retrieved data on the participants’ basic characteristics such as age, body mass index (BMI; kg/m^2^), FFM (kg), serum 25-hydroxy vitamin D level (ng/mL), daily calcium intake (mg), daily nutritional intake (kcal), water intake per kilogram body weight (g/kg), smoking status (non-smoker, past smoker, or current smoker), alcohol consumption status (more than one drink per month for the past 1 year), education level (elementary school or lower, middle school, high school, or college graduate or higher), average monthly household income (10,000 KRW), occupation and survey year.

The participants’ physical activity (PA) level was measured in metabolic equivalents (METs) according to guidelines for the processing and analysis of International Physical Activity Questionnaire data [18]. Serum vitamin D levels were classified as insufficient or sufficient based on a cutoff of 20 ng/mL [19] and daily calcium intake was classified as unsatisfactory or satisfactory based on a recommended daily intake of 700 mg [20].

We categorized the participants as follows: group 1 (serum vitamin D level < 20 ng/mL and daily calcium intake < 700 mg); group 2 (serum vitamin D level ≥ 20 ng/mL and daily calcium intake < 700 mg); group 3 (serum vitamin D level < 20 ng/mL and daily calcium intake ≥ 700 mg); and group 4 (serum vitamin D level ≥ 20 ng/mL and daily calcium intake ≥ 700 mg).

### 2.3. Statistical Analysis

Statistical analyses were performed using STATA version 14.0 (StataCorp., College Station, TX, USA) and the level of significance was set at *p* < 0.05. Sampling for the KNHANES was performed using two-stage stratified cluster sampling rather than simple random sampling and we weighted the data during analysis to reflect this. Linear regression analysis and the chi-square test were used to compare and analyze the basic characteristics of the participants by sex and group.

Linear regression analysis was used to analyze the differences in FFM among the four groups. We performed multiple linear regression analysis with adjustments for age, BMI (<25 vs. ≥25 kg/m^2^), daily nutritional intake, water intake per kilogram body weight, smoking status, alcohol consumption status, PA level, education level, average monthly household income, occupation and survey year.

A sensitivity analysis was performed by changing the serum vitamin D level cutoff to 10 ng/mL or 30 ng/mL and changing recommended daily calcium intake to 800 mg or 1000 mg. We also conducted an analysis with serum vitamin D levels and daily calcium intake values as continuous variables.

## 3. Results

### 3.1. Basic Characteristics of the Participants by Sex

Table 1 shows the basic characteristics of the participants by sex. The average age of the 14,444 participants was 44.77 years old and 59.46% of them were women. The average FFM of all the participants was 43.03 kg, with higher values in men than in women (51.14 ± 0.13 vs. 35.95 ± 0.078 kg, *p* < 0.001). The mean serum vitamin D level of all the participants was 18.00 ng/mL, with higher levels in men than in women (19.34 ± 0.20 vs. 16.83 ± 0.15 ng/mL, *p* < 0.001). The average daily calcium intake of all the participants was 0.51 g, with higher levels in men than in women (0.57 ± 0.006 vs. 0.45 ± 0.005 g, *p* < 0.001). Higher values in men than in women were also observed for BMI, total energy intake, water intake per kilogram body weight, PA level and average monthly household income. Furthermore, the proportion of current smokers, alcohol drinkers (≥1 time/month), highly educated participants (≥college) and participants with occupation was also higher for men than for women.

### 3.2. Basic Characteristics of the Participants by Group

In men, the FFM, water intake per kilogram body weight, PA level and the proportion of participants with a BMI ≥ 25 kg/m^2^ and occupation were the highest in group 4. The total energy intake, average monthly household income and proportion of highly educated participants (≥college) were the highest in group 3. The proportion of current smokers was the highest in group 1 (Table 2).

In women, the PA level was the highest in group 4. The total energy intake, water intake per kilogram body weight, average monthly household income and proportion of highly educated participants (≥college) and participants with occupation were the highest in group 3. The proportion of participants with a BMI ≥ 25 kg/m^2^ was the highest in group 2. The proportion of alcohol drinkers (≥1 time/month) was the highest in group 1 (Table 3).

### 3.3. Differences in Fat Free Mass According to Serum Vitamin D Level and Daily Calcium Intake

In men, after adjusting for potential confounding factors the FFM of group 2, group 3 and group 4 was 0.50 kg (95% confidence interval (CI), 0.084–0.92), 0.78 kg (95% CI, 0.26–1.29) and 1.58 kg (95% CI, 0.95–2.21) higher than that of group 1, respectively (Table 4).

In women, after adjusting for potential confounding factors the FFM of group 4 was 0.55 kg (95% CI, 0.002–1.09) higher than that of group 1 (Table 5).

### 3.4. Sensitivity Analysis

Linear regression analyses of changes in FFM by group were performed with the following values: when the serum vitamin D level cutoff was 10 ng/mL and the recommended daily calcium intake was 700 mg (Table S1); serum vitamin D level cutoff of 30 ng/mL and recommended daily calcium intake of 700 mg (Table S2); serum vitamin D level cutoff of 10 ng/mL and recommended daily calcium intake of 800 mg (Table S3); serum vitamin D level cutoff of 20 ng/mL and recommended daily calcium intake of 800 mg (Table S4); serum vitamin D level cutoff of 30 ng/mL and recommended daily calcium intake of 800 mg (Table S5); serum vitamin D level cutoff of 10 ng/mL and recommended daily calcium intake of 1000 mg (Table S6); serum vitamin D level cutoff of 20 ng/mL and recommended daily calcium intake of 1000 mg (Table S7); serum vitamin D level cutoff of 30 ng/mL and recommended daily calcium intake of 1000 mg (Table S8).

In men, the FFM of group 2, group 3 and group 4 was higher than that of group 1 (Tables S1 and S7). The FFM of group 2 and group 4 was higher than that of group 1 (Tables S3, S4 and S6). The FFM of group 3 and group 4 was higher than that of group 1 (Tables S2, S5 and S8).

In women, the FFM of group 2 and group 4 was higher than that of group 1 (Tables S1, S3 and S6). The FFM of group 3 and group 4 was higher than that of group 1 (Table S5). The FFM of group 4 was higher than that of group 1 (Tables S2 and S4). For certain cutoffs, there was no significant difference in the FFM among the groups (Tables S7 and S8).

When the serum vitamin D level and daily calcium intake were analyzed as continuous variables (Table S9), in men, a 1 ng/mL increase in serum vitamin D level was associated with a 0.061 kg increase in FFM (95% CI 0.035–0.086) and a 1 g increase in daily calcium intake was associated with a 1.13 kg increase in FFM (95% CI 0.59–1.68). In women, a 1 ng/mL increase in serum vitamin D level was associated with a 0.023 kg increase in FFM (95% CI 0.003–0.043) and a 1 g increase in daily calcium intake was associated with a 0.62 kg increase in FFM (95% CI, 0.067–1.16).

## 4. Discussion

In this study, we found that high serum vitamin D level and daily calcium intake were associated with a high FFM.

A recent study reported that vitamin D insufficiency, along with high BMI, was related to paraspinal muscle atrophy in postmenopausal women [21]. However, there was no evidence that vitamin D supplementation had beneficial effects on muscle health, according to a recent meta-analysis [22]. A recent study reported that low calcium intake may be a predictor of muscle loss in women aged over 50 years [13].

We derived these results by analyzing the relationship between these variables by studying them using various cutoffs of vitamin D level and calcium intake and considering them as continuous variables. In recent years, there has been increasing interest in vitamin D and calcium and the same is true for sarcopenia. However, the serum vitamin D level and daily calcium intake required to sustain muscle health has not been clearly determined. The clinical practice guidelines formulated by the Endocrine Society Task Force on Vitamin D [23] defined vitamin D deficiency as a serum vitamin D level less than 50 nmol/L (20 ng/mL) and a Korean guideline also defines vitamin D deficiency in this manner [19]. In another study, a daily calcium intake of at least 668 mg/day was found to be sufficient to maintain bone mass [20]. Therefore, we used a serum vitamin D level cutoff of 20 ng/mL and recommended daily calcium intake of 700 mg. However, a sensitivity analysis performed by varying these cutoffs yielded some significant results. Moreover, in women, the results of the analyses of groups created using the abovementioned cutoffs were not significant in many cases, but they were significant when these variables were analyzed as continuous variables. Therefore, the serum vitamin D level or daily calcium intake required to sustain sufficient muscle health should be further explored.

The mechanisms by which vitamin D and calcium act on body fat and muscle are unclear.

In 1972, it was suggested that muscle and fat could be important reservoirs for vitamin D [24]. It was found that injected radioactive cholecalciferol was rapidly distributed from the serum and that adipose tissue and voluntary muscle were the principal sites of vitamin D storage in humans. In addition, vitamin D receptors are present in human skeletal muscle tissue [25]. Through those receptors, vitamin D may promote the expression of actin, troponin C and components of the sarcoplasmic reticulum [26]. It can also stimulate fatty acid oxidation and mitochondrial metabolism [27]. Vitamin D supplementation has been found to reduce weight gain and fat accumulation in mice, possibly due to the robust induction of genes involved in fatty acid oxidation and mitochondrial biogenesis and function. Body fat may inhibit vitamin D synthesis via inflammatory mechanisms mediated by leptin and interleukin-6 [28]. In a longitudinal study involving 859 participants, the associations between changes in serum vitamin D levels and body adiposity were studied over 2.6 years. Those who recovered from vitamin D deficiency had a lower body fat percentage and lower serum leptin levels than those who did not recover from vitamin D deficiency, which suggests that there may be an association between adiposity and vitamin D levels mediated by leptin. Body fat has been found to be inversely associated with serum vitamin D levels in healthy black and white women [29]. In addition, healthy, premenopausal, African American women with a low calcium and vitamin D intake were found to be likely to have excessive adiposity [30].

Calcium enhances adipose tissue apoptosis through molecular mechanisms such as uncoupling protein 2 expression [31]. A high dietary calcium intake without caloric restriction reduces adipocyte triglyceride accumulation and results in a net reduction of fat mass in both mice and humans. This indicates that calcium can reduce not only adipocyte size but also adipocyte number. People who increase their dietary calcium intake tend to excrete increased amounts of fat and energy in their feces [32]. It has been reported that the total amount of fat and energy excreted in the feces is higher in the high calcium and normal protein diet than in the low calcium and normal protein diet for one week. Calcium intake has also been linked to appetite regulation in humans [33]. A previous review reported that the intake of various foods and nutrients can be regulated by calcium.

In this study, high total energy intake was found in those with satisfactory daily calcium intake and obesity was more prevalent in those with sufficient serum vitamin D levels. Although obesity itself may increase FFM, daily calcium intake and serum vitamin D levels were significantly associated with FFM even after controlling for obesity and total energy intake as confounding variables.

The limitations of this study are as follows: First, because this was a cross-sectional study, we could not evaluate the cause–effect relationship of FFM with serum vitamin D level and calcium intake. Therefore, randomized controlled trials should be conducted to confirm whether increasing the serum vitamin D level and daily calcium intake can actually increase the FFM. Second, although the KNHANES has been conducted since 1998, fat mass and FFM were measured only from 2008 to 2011. Therefore, these results may not reflect the latest data. Considering the increasing interest in sarcopenia, a national survey should be conducted to measure the body composition of the population. Third, a seasonal variation in vitamin D levels has been reported [34], suggesting that the time of sample collection can be important, but this was not considered in this analysis. These limitations should be considered when designing future studies.

## 5. Conclusions

In this study, high serum vitamin D levels and daily calcium intake were associated with a high FFM. Vitamin D [35] and calcium [36] are known to have various positive effects in patients with metabolic disorders. Therefore, we can expect to be helpful in the prevention of metabolic diseases in adults through the improvement of nutrition intake as well as indirectly through the change in muscle mass.

## Figures and Tables

**Table 1:** Basic characteristics of the participants by sex.

Characteristics		Total (*n* = 14,444)	Male (*n* = 5856)	Female (*n* = 8588)	*p*-Value ^a^
Age, years		44.77 ± 0.26	44.40 ± 0.32	45.09 ± 0.27	0.019
25-hydroxy vitamin D, ng/mL		18.00 ± 0.16	19.34 ± 0.20	16.83 ± 0.15	<0.001
Calcium intake, mg		507.30 ± 3.91	573.09 ± 5.70	449.81 ± 4.65	<0.001
Group					<0.001
	1	7550 (52.27)	2363 (40.35)	5187 (60.40)	
	2	4130 (28.59)	1994 (34.05)	2136 (24.87)	
	3	1741 (12.05)	843 (14.40)	898 (10.46)	
	4	1023 (7.08)	656 (11.20)	367 (4.27)	
Whole body total fat-free mass, kg		43.03 ± 0.11	51.14 ± 0.13	35.95 ± 0.078	<0.001
Body mass index, kg/m^2^		23.58 ± 0.040	24.02 ± 0.055	23.20 ± 0.055	<0.001
Body mass index, kg/m^2^					<0.001
	<25	9964 (68.98)	3808 (65.03)	6156 (71.68)	
	≥25	4480 (31.02)	2048 (34.97)	2432 (28.32)	
Nutritional intake					
	Total energy intake, kcal/day	1973.62 ± 9.70	2322.87 ± 14.29	1688.46 ± 9.29	<0.001
	Water intake/body weight, g/kg/day	15.54 ± 0.13	16.29 ± 0.17	14.89 ± 0.15	<0.001
Smoking					<0.001
	None	9132 (63.22)	1293 (22.08)	7839 (91.28)	
	Past	2486 (17.21)	2165 (36.97)	321 (3.74)	
	Current	2826 (19.57)	2398 (40.95)	428 (4.98)	
Alcohol drinking					<0.001
	<1 time/month	6785 (46.97)	1527 (26.08)	5258 (61.22)	
	≥1 time/month	7659 (53.03)	4329 (73.92)	3330 (38.78)	
Physical activity, MET-min/week		2548.57 ± 38.80	2863.09 ± 56.27	2263.16 ± 40.99	<0.001
Education					<0.001
	≤Elementary school	3868 (26.78)	1132 (19.33)	2736 (31.86)	
	Middle school	1685 (11.67)	797 (13.61)	888 (10.34)	
	High school	4925 (34.10)	2073 (35.40)	2852 (33.21)	
	≥College	3966 (27.46)	1854 (31.66)	2112 (24.59)	
Average monthly household income, KRW		325.88 ± 4.79	330.78 ± 5.51	321.60 ± 5.02	0.034
Occupation					<0.001
	No	5769 (39.94)	1406 (24.01)	4363 (50.80)	
	Yes	8675 (60.06)	4450 (75.99)	4225 (49.20)	
Survey year					0.089
	2008	2613 (18.09)	1007 (17.20)	1606 (18.70)	
	2009	5497 (38.06)	2275 (38.85)	3222 (37.52)	
	2010	4462 (30.89)	1826 (31.18)	2636 (30.69)	
	2011	1872 (12.96)	748 (12.77)	1124 (13.09)	

MET, metabolic equivalent task; KRW, Korea Republic Won. Data are presented as means ± standard error for continuous variables and numbers (%) for categorical variables. Group 1: 25-hydroxy vitamin D < 20 ng/mL and calcium intake < 700 mg. Group 2: 25-hydroxy vitamin D ≥ 20 ng/mL and calcium intake < 700 mg. Group 3: 25-hydroxy vitamin D < 20 ng/mL and calcium intake ≥ 700 mg. Group 4: 25-hydroxy vitamin D ≥ 20 ng/mL and calcium intake ≥ 700 mg. ^a^ *p*-value from linear regression analysis for continuous variables or χ^2^ test for categorical variables, comparing differences between two groups.

**Table 2:** Comparison of basic characteristics by group in men.

Characteristics		Group 1 (*n* = 2363)	Group 2 (*n* = 1994)	Group 3 (*n* = 843)	Group 4 (*n* = 656)	*p*-Value ^a^
Age, years		41.83 ± 0.43	48.68 ± 0.52	41.63 ± 0.54	46.92 ± 0.72	<0.001
25-hydroxy vitamin D, ng/mL		14.66 ± 0.09	26.06 ± 0.17	14.88 ± 0.15	26.11 ± 0.25	<0.001
Calcium intake, mg		411.66 ± 3.85	418.59 ± 4.53	991.13 ± 13.66	1026.84 ± 20.17	<0.001
Whole body total fat-free mass, kg		50.95 ± 0.18	50.44 ± 0.20	52.13 ± 0.27	52.40 ± 0.33	<0.001
Body mass index, kg/m^2^		24.05 ± 0.10	23.86 ± 0.09	24.06 ± 0.12	24.27 ± 0.14	0.078
Body mass index, kg/m^2^						0.030
	<25	1542 (65.26)	1335 (66.95)	524 (62.16)	407 (62.04)	
	≥25	821 (34.74)	659 (33.05)	319 (37.84)	249 (37.96)	
Nutritional intake						
	Total energy intake, kcal/day	2140.20 ± 20.49	2139.23 ± 19.97	2824.45 ± 34.65	2816.42 ± 42.94	<0.001
	Water intake/body weight, g/kg/day	14.18 ± 0.24	14.68 ± 0.23	21.30 ± 0.43	21.74 ± 0.46	<0.001
Smoking						<0.001
	None	537 (22.73)	390 (19.56)	208 (24.67)	158 (24.09)	
	Past	798 (33.77)	824 (41.32)	279 (33.10)	264 (40.24)	
	Current	1028 (43.50)	780 (39.12)	356 (42.23)	234 (35.67)	
Alcohol drinking						0.096
	<1 time/month	657 (27.80)	491 (24.62)	214 (25.39)	165 (25.15)	
	≥1 time/month	1706 (72.20)	1503 (75.38)	629 (74.61)	491 (74.85)	
Physical activity, MET-min/week		2566.11 ± 74.84	3262.39 ± 100.46	2551.85 ± 115.03	3420.64 ± 168.62	<0.001
Education						<0.001
	≤Elementary school	372 (15.74)	560 (28.08)	73 (8.66)	127 (19.36)	
	Middle school	299 (12.65)	324 (16.25)	81 (9.61)	93 (14.18)	
	High school	890 (37.66)	628 (31.49)	327 (38.79)	228 (34.76)	
	≥College	802 (33.94)	482 (24.17)	362 (42.94)	208 (31.71)	
Average monthly household income, KRW		325.29 ± 8.55	308.49 ± 8.18	371.72 ± 11.01	353.41 ± 11.84	<0.001
Occupation						<0.001
	No	655 (27.72)	453 (22.72)	170 (20.17)	128 (19.51)	
	Yes	1708 (72.28)	1541 (77.28)	673 (79.83)	528 (80.49)	
Survey year						<0.001
	2008	245 (10.37)	544 (27.28)	67 (7.95)	151 (23.02)	
	2009	1005 (42.53)	745 (37.36)	288 (34.16)	237 (36.13)	
	2010	734 (31.06)	558 (27.98)	312 (37.01)	222 (33.84)	
	2011	379 (16.04)	147 (7.37)	176 (20.88)	46 (7.01)	

MET, metabolic equivalent task; KRW, Korea Republic Won. Data are presented as means ± standard error for continuous variables and numbers (%) for categorical variables. Group 1: 25-hydroxy vitamin D < 20 ng/mL and calcium intake < 700 mg. Group 2: 25-hydroxy vitamin D ≥ 20 ng/mL and calcium intake < 700 mg. Group 3: 25-hydroxy vitamin D < 20 ng/mL and calcium intake ≥ 700 mg. Group 4: 25-hydroxy vitamin D ≥ 20 ng/mL and calcium intake ≥ 700 mg. ^a^ *p*-value from linear regression analysis for continuous variables or χ^2^ test for categorical variables, comparing differences between two groups.

**Table 3:** Comparison of basic characteristics by group in women.

Characteristics		Group 1 (*n* = 5187)	Group 2 (*n* = 2136)	Group 3 (*n* = 898)	Group 4 (*n* = 367)	*p*-Value ^a^
Age, years		43.80 ± 0.32	49.54 ± 0.53	42.80 ± 0.54	47.47 ± 1.06	<0.001
25-hydroxy vitamin D, ng/mL		13.77 ± 0.08	25.55 ± 0.15	14.22 ± 0.14	24.97 ± 0.25	<0.001
Calcium intake, mg		353.63 ± 2.68	360.25 ± 4.31	989.87 ± 20.86	1003.28 ± 22.08	<0.001
Whole body total fat-free mass, kg		35.89 ± 0.10	35.97 ± 0.12	36.05 ± 0.19	36.50 ± 0.30	0.249
Body mass index, kg/m^2^		23.19 ± 0.07	23.35 ± 0.10	22.85 ± 0.15	23.49 ± 0.24	0.022
Body mass index, kg/m^2^						<0.001
	<25	3749 (72.28)	1471 (68.87)	681 (75.84)	255 (69.48)	
	≥25	1438 (27.72)	665 (31.13)	217 (24.16)	112 (30.52)	
Nutritional intake						
	Total energy intake, kcal/day	1572.87 ± 10.54	1574.67 ± 16.35	2231.85 ± 31.68	2173.74 ± 46.32	<0.001
	Water intake/body weight, g/kg/day	13.40 ± 0.15	13.82 ± 0.25	22.94 ± 0.49	22.65 ± 0.71	<0.001
Smoking						0.555
	None	4716 (90.92)	1966 (92.04)	817 (90.98)	340 (92.64)	
	Past	195 (3.76)	77 (3.60)	35 (3.90)	14 (3.81)	
	Current	276 (5.32)	93 (4.35)	46 (5.12)	13 (3.54)	
Alcohol drinking						0.050
	<1 time/month	3128 (60.30)	1360 (63.67)	541 (60.24)	229 (62.40)	
	≥1 time/month	2059 (39.70)	776 (36.33)	357 (39.76)	138 (37.60)	
Physical activity, MET-min/week		2108.05 ± 43.36	2616.54 ± 89.18	2136.49 ± 97.36	3049.05 ± 242.17	<0.001
Education						<0.001
	≤Elementary school	1505 (29.01)	955 (44.71)	161 (17.93)	115 (31.34)	
	Middle school	493 (9.50)	252 (11.80)	97 (10.80)	46 (12.53)	
	High school	1851 (35.69)	564 (26.40)	323 (35.97)	114 (31.06)	
	≥College	1338 (25.80)	365 (17.09)	317 (35.30)	92 (25.07)	
Average monthly household income, KRW		333.14 ± 5.94	281.27 ± 6.95	336.61 ± 10.84	317.52 ± 16.50	<0.001
Occupation						0.804
	No	2645 (50.99)	1085 (50.80)	443 (49.33)	190 (51.77)	
	Yes	2542 (49.01)	1051 (49.20)	455 (50.67)	177 (48.23)	
Survey year						<0.001
	2008	735 (14.17)	654 (30.62)	112 (12.47)	105 (28.61)	
	2009	2013 (38.81)	787 (36.84)	311 (34.63)	111 (30.25)	
	2010	1627 (31.37)	562 (26.31)	336 (37.42)	111 (30.25)	
	2011	812 (15.65)	133 (6.23)	139 (15.48)	40 (10.90)	

MET, metabolic equivalent task; KRW, Korea Republic Won. Data are presented as means ± standard error for continuous variables and numbers (%) for categorical variables. Group 1: 25-hydroxy vitamin D < 20 ng/mL and calcium intake < 700 mg. Group 2: 25-hydroxy vitamin D ≥ 20 ng/mL and calcium intake < 700 mg. Group 3: 25-hydroxy vitamin D < 20 ng/mL and calcium intake ≥ 700 mg. Group 4: 25-hydroxy vitamin D ≥ 20 ng/mL and calcium intake ≥ 700 mg. ^a^ *p*-value from linear regression analysis for continuous variables or χ^2^ test for categorical variables, comparing differences between two groups.

**Table 4:** Linear regression analysis of changes in whole body total fat-free mass by group in men.

Characteristics		Crude	Age-Adjusted	Multivariable ^a^
Age, years		−0.14 (−0.15, −0.13)		−0.11 (−0.13, −0.095)
Group				
	1 (*n* = 2363)			
	2 (*n* = 1994)	−0.51 (−1.02, −0.003)	0.46 (−0.016, 0.95)	0.50 (0.084, 0.92)
	3 (*n* = 843)	1.18 (0.56, 1.79)	1.15 (0.54, 1.75)	0.78 (0.26, 1.29)
	4 (*n* = 656)	1.45 (0.76, 2.14)	2.18 (1.48, 2.88)	1.58 (0.95, 2.21)
Body mass index, kg/m^2^				
	<25			
	≥25	7.40 (6.99, 7.81)	7.38 (6.99, 7.77)	6.84 (6.47, 7.21)
Nutritional intake				
	Total energy intake, kcal/day	0.001 (0.001, 0.002)	0.001 (0.0008, 0.001)	0.001 (0.001, 0.002)
	Water intake/body weight, g/kg/day	−0.030 (−0.050, −0.009)	−0.048 (−0.068, −0.028)	−0.12 (−0.14, −0.10)
Smoking				
	None			
	Past	−0.72 (−1.36, −0.082)	1.00 (0.34, 1.66)	0.42 (−0.082, 0.92)
	Current	0.13 (−0.43, 0.69)	0.47 (−0.092, 1.03)	0.27 (−0.17, 0.71)
Alcohol drinking				
	<1 time/month			
	≥1 time/month	1.33 (0.86, 1.81)	0.66 (0.21, 1.11)	0.39 (0.004, 0.77)
Physical activity, MET-min/week		0.0002 (0.00008, 0.0002)	0.0002 (0.00009, 0.0003)	0.0001 (0.00006, 0.0002)
Education				
	≤Elementary school			
	Middle school	2.46 (1.71, 3.21)	1.67 (0.98, 2.36)	1.04 (0.40, 1.67)
	High school	4.79 (4.21, 5.37)	2.22 (1.58, 2.87)	1.45 (0.87, 2.03)
	≥College	5.28 (4.72, 5.85)	2.79 (2.16, 3.42)	1.66 (1.07, 2.25)
Average monthly household income, KRW		0.004 (0.003, 0.005)	0.003 (0.002, 0.004)	0.002 (0.001, 0.003)
Occupation				
	No			
	Yes	2.08 (1.56, 2.60)	1.77 (1.27, 2.27)	0.55 (0.15, 0.95)
Survey year				
	2008			
	2009	−0.40 (−1.09, 0.29)	−0.43 (−1.10, 0.23)	−0.51 (−1.07, 0.045)
	2010	−1.03 (−1.76, −0.30)	−0.99 (−1.66, −0.31)	−1.32 (−1.93, −0.71)
	2011	0.21 (−0.76, 1.17)	0.22 (−0.64, 1.08)	−0.050 (−0.83, 0.73)

MET, metabolic equivalent task; KRW, Korea Republic Won. Data are presented as beta coefficient (95% confidence interval). Group 1: 25-hydroxy vitamin D < 20 ng/mL and calcium intake < 700 mg. Group 2: 25-hydroxy vitamin D ≥ 20 ng/mL and calcium intake < 700 mg. Group 3: 25-hydroxy vitamin D < 20 ng/mL and calcium intake ≥ 700 mg. Group 4: 25-hydroxy vitamin D ≥ 20 ng/mL and calcium intake ≥ 700 mg. ^a^ Multivariable model adjusted for age, body mass index status, total energy intake, water intake per body weight, smoking, alcohol drinking, physical activity, education, income, occupation and survey year.

**Table 5:** Linear regression analysis of changes in whole body total fat-free mass by group in women.

Characteristics		Crude	Age-Adjusted	Multivariable ^a^
Age, years		−0.029 (−0.037, −0.021)		−0.032 (−0.043, −0.022)
Group				
	1 (*n* = 5187)			
	2 (*n* = 2136)	0.083 (−0.20, 0.37)	0.25 (−0.031, 0.54)	0.16 (−0.10, 0.43)
	3 (*n* = 898)	0.16 (−0.24, 0.57)	0.13 (−0.27, 0.54)	0.29 (−0.10, 0.68)
	4 (*n* = 367)	0.62 (−0.007, 1.25)	0.73 (0.11, 1.35)	0.55 (0.002, 1.09)
Body mass index, kg/m^2^				
	<25			
	≥25	4.99 (4.72, 5.26)	5.41 (5.14, 5.68)	5.15 (4.86, 5.43)
Nutritional intake				
	Total energy intake, kcal/day	0.0005 (0.0003, 0.0007)	0.0004 (0.0002, 0.0006)	0.0009 (0.0007, 0.001)
	Water intake/body weight, g/kg/day	−0.057 (−0.070, −0.044)	−0.068 (−0.081, −0.055)	−0.072 (−0.089, −0.056)
Smoking				
	None			
	Past	0.65 (−0.10, 1.41)	0.56 (−0.18, 1.31)	0.57 (−0.14, 1.27)
	Current	−0.10 (−0.66, 0.46)	−0.21 (−0.76, 0.35)	−0.34 (−0.82, 0.14)
Alcohol drinking				
	<1 time/month			
	≥1 time/month	0.72 (0.47, 0.97)	0.53 (0.28, 0.78)	0.42 (0.21, 0.64)
Physical activity, MET-min/week		0.0002 (0.0001, 0.0002)	0.0002 (0.0002, 0.0002)	0.0002 (0.0001, 0.0002)
Education				
	≤Elementary school			
	Middle school	1.21 (0.77, 1.65)	0.91 (0.46, 1.36)	0.94 (0.54, 1.35)
	High school	1.33 (1.01, 1.66)	0.70 (0.27, 1.13)	1.29 (0.88, 1.70)
	≥College	0.59 (0.29, 0.90)	−0.12 (−0.60, 0.36)	1.05 (0.60, 1.51)
Average monthly household income, KRW		0.0004 (−0.00009, 0.0009)	−0.0000003 (−0.0005, 0.0005)	0.0005 (0.00005, 0.0009)
Occupation				
	No			
	Yes	0.44 (0.20, 0.69)	0.36 (0.12, 0.60)	0.10 (−0.11, 0.32)
Survey year				
	2008			
	2009	0.080 (−0.33, 0.49)	0.10 (−0.31, 0.52)	−0.017 (−0.39, 0.35)
	2010	−0.77 (−1.19, −0.35)	−0.73 (−1.15, −0.30)	−0.83 (−1.25, −0.42)
	2011	0.18 (−0.34, 0.69)	0.21 (−0.30, 0.72)	−0.14 (−0.62, 0.33)

MET, metabolic equivalent task; KRW, Korea Republic Won. Data are presented as beta coefficient (95% confidence interval). Group 1: 25-hydroxy vitamin D < 20 ng/mL and calcium intake < 700 mg. Group 2: 25-hydroxy vitamin D ≥ 20 ng/mL and calcium intake < 700 mg. Group 3: 25-hydroxy vitamin D < 20 ng/mL and calcium intake ≥ 700 mg. Group 4: 25-hydroxy vitamin D ≥ 20 ng/mL and calcium intake ≥ 700 mg. ^a^ Multivariable model adjusted for age, body mass index status, total energy intake, water intake per body weight, smoking, alcohol drinking, physical activity, education, income, occupation and survey year.

## Data Availability

The data presented in this study are publicly available in the Korea National Health and Nutrition Examination Survey database at https://knhanes.kdca.go.kr/knhanes/sub03/sub03_02_05.do (accessed on 01 September 2021) and https://knhanes.kdca.go.kr/knhanes/eng/index.do (accessed on 01 September 2021).

## Supplementary Materials

The following are available online at https://www.mdpi.com/article/10.3390/jcm10225428/s1. Table S1: Linear regression analysis of changes in whole body total fat-free mass by group (serum vitamin D level cutoff, 10 ng/mL; recommended daily calcium intake, 700 mg), Table S2: Linear regression analysis of changes in whole body total fat-free mass by group (serum vitamin D level cutoff, 30 ng/mL; recommended daily calcium intake, 700 mg), Table S3: Linear regression analysis of changes in whole body total fat-free mass by group (serum vitamin D level cutoff, 10 ng/mL; recommended daily calcium intake, 800 mg), Table S4: Linear regression analysis of changes in whole body total fat-free mass by group (serum vitamin D level cutoff, 20 ng/mL; recommended daily calcium intake, 800 mg), Table S5: Linear regression analysis of changes in whole body total fat-free mass by group (serum vitamin D level cutoff, 30 ng/mL; recommended daily calcium intake, 800 mg), Table S6: Linear regression analysis of changes in whole body total fat-free mass by group (serum vitamin D level cutoff, 10 ng/mL; recommended daily calcium intake, 1000 mg), Table S7: Linear regression analysis of changes in whole body total fat-free mass by group (serum vitamin D level cutoff, 20 ng/mL; recommended daily calcium intake, 1000 mg), Table S8: Linear regression analysis of changes in whole body total fat-free mass by group (serum vitamin D level cutoff, 30 ng/mL; recommended daily calcium intake, 1000 mg), Table S9, Linear regression analysis of changes in whole body total fat-free mass according to serum vitamin D level and daily calcium intake.
