# Supplementary material for: Differences in Fat-Free Mass According to Serum Vitamin D Level and Calcium Intake: Korea National Health and Nutrition Examination Survey 2008–2011
Source: J Clin Med. 2021 Nov 20;10(22):5428. doi: 10.3390/jcm10225428 (PMC8623334; doi:10.3390/jcm10225428)
Supplement: Supplementary file 1 [file jcm-10-05428-s001.zip › jcm-1417619-supplementary.pdf]

Journal of Clinical Medicine

Table S1. Linear regression analysis of changes in whole body total fat−free mass by group (serum vitamin D level cutoff: 10 ng/mL, recommended daily calcium intake: 700 mg)

**Characteristics**		Men	Women
Age, years		−0.11 (−0.12, −0.093)	−0.032 (−0.043, −0.022)
Group			
	1 (n = 178/709)		
	2 (n = 4,179/6,614)	2.18 (1.34, 3.02)	0.71 (0.17, 1.26)
	3 (n = 67/93)	2.54 (0.88, 4.19)	0.78 (−0.56, 2.12)
	4 (n = 1,432/1,172)	3.00 (2.10, 3.90)	0.98 (0.36, 1.60)
Body mass index, kg/m^2			
	< 25		
	≥ 25	6.82 (6.45, 7.19)	5.14 (4.85, 5.43)
Nutritional intake			
	Total energy intake, kcal/day	0.001 (0.001, 0.002)	0.0009 (0.0007, 0.001)
	Water intake/body weight, g/kg/day	−0.12 (−0.14, −0.10)	−0.072 (−0.088, −0.056)
Smoking			
	None		
	Past	0.42 (−0.075, 0.92)	0.57 (−0.14, 1.28)
	Current	0.29 (−0.16, 0.73)	−0.34 (−0.82, 0.15)
Alcohol drinking			
	<1 time/month		
	≥1 time/month	0.40 (0.029, 0.78)	0.40 (0.19, 0.61)
Physical activity, MET-min/week		0.0001 (0.00007, 0.0002)	0.0002 (0.0001, 0.0002)
Education			
	≤Elementary school		
	Middle school	1.00 (0.36, 1.64)	0.94 (0.54, 1.35)
	High school	1.41 (0.84, 1.99)	1.29 (0.89, 1.70)
	≥College	1.60 (1.00, 2.19)	1.05 (0.60, 1.50)
Average monthly household income, 10000 KRW		0.002 (0.001, 0.003)	0.0005 (0.00005, 0.001)
Occupation			
	No		
	Yes	0.51 (0.11, 0.92)	0.10 (−0.12, 0.32)
Survey year			
	2008		
	2009	−0.63 (−1.19, −0.076)	−0.023 (−0.38, 0.338)
	2010	−1.41 (−2.02, −0.80)	−0.83 (−1.25, −0.42)
	2011	−0.19 (−0.96, 0.57)	−0.13 (−0.60, 0.34)

MET, Metabolic Equivalent Task; KRW, Korea Republic Won

Data are presented as beta coefficient (95% confidence interval).

Group 1: 25-hydroxy vitamin D < 10 ng/mL and calcium intake < 700 mg

Group 2: 25-hydroxy vitamin D ≥ 10 ng/mL and calcium intake < 700 mg

Group 3: 25-hydroxy vitamin D < 10 ng/mL and calcium intake ≥ 700 mg

Group 4: 25-hydroxy vitamin D ≥ 10 ng/mL and calcium intake ≥ 700 mg

[a]Multivariable model adjusted for age, body mass index status, total energy intake, water intake per body weight, smoking, alcohol drinking, physical activity, education, income, occupation, and survey year.

Journal of
Clinical Medicine

MDPI

Table S2. Linear regression analysis of changes in whole body total fat-free mass by group (serum vitamin D level cutoff: 30 ng/mL, recommended daily calcium intake: 700 mg)

**Characteristics**		Men	Women
Age, years		−0.11 (−0.12, −0.092)	−0.032 (−0.042, −0.021)
Group			
	1 (n = 3,915/6,940)		
	2 (n = 442/383)	0.19 (−0.42, 0.80)	0.12 (−0.38, 0.62)
	3 (n = 1,359/1,210)	0.81 (0.38, 1.23)	0.27 (−0.06, 0.60)
	4 (n = 140/55)	2.08 (0.98, 3.18)	1.63 (0.47, 2.78)
Body mass index, kg/m^2			
	< 25		
	≥ 25	6.84 (6.47, 7.21)	5.15 (4.87, 5.44)
Nutritional intake			
	Total energy intake, kcal/day	0.001 (0.001, 0.002)	0.0009 (0.0007, 0.001)
	Water intake/body weight, g/kg/day	−0.12 (−0.14, −0.10)	−0.072 (−0.088, −0.055)
Smoking			
	None		
	Past	0.42 (−0.080, 0.92)	0.57 (−0.13, 1.28)
	Current	0.24 (−0.20, 0.68)	−0.36 (−0.84, 0.13)
Alcohol drinking			
	<1 time/month		
	≥1 time/month	0.43 (0.046, 0.81)	0.42 (0.21, 0.64)
Physical activity, MET-min/week		0.0001 (0.00007, 0.0002)	0.0002 (0.0001, 0.0002)
Education			
	≤Elementary school		
	Middle school	1.02 (0.38, 1.66)	0.94 (0.53, 1.34)
	High school	1.42 (0.84, 2.01)	1.29 (0.88, 1.69)
	≥College	1.61 (1.01, 2.21)	1.04 (0.59, 1.49)
Average monthly household income, 10000 KRW		0.002 (0.001, 0.003)	0.0005 (0.00005, 0.001)
Occupation			
	No		
	Yes	0.58 (0.18, 0.98)	0.10 (−0.12, 0.32)
Survey year			
	2008		
	2009	−0.63 (−1.18, −0.073)	−0.039 (−0.41, 0.329)
	2010	−1.44 (−2.04, −0.83)	−0.86 (−1.27, −0.45)
	2011	−0.24 (−1.01, 0.53)	−0.18 (−0.64, 0.29)

MET, Metabolic Equivalent Task; KRW, Korea Republic Won

Data are presented as beta coefficient (95% confidence interval).

Journal of
Clinical Medicine

Group 1: 25-hydroxy vitamin D < 30 ng/mL and calcium intake < 700 mg

Group 2: 25-hydroxy vitamin D ≥ 30 ng/mL and calcium intake < 700 mg

Group 3: 25-hydroxy vitamin D < 30 ng/mL and calcium intake ≥ 700 mg

Group 4: 25-hydroxy vitamin D ≥ 30 ng/mL and calcium intake ≥ 700 mg

[a]Multivariable model adjusted for age, body mass index status, total energy intake, water intake per body weight, smoking, alcohol drinking, physical activity, education, income, occupation, and survey year.

Journal of
Clinical Medicine

MDPI

Table S3. Linear regression analysis of changes in whole body total fat-free mass by group (serum vitamin D level cutoff: 10 ng/mL, recommended daily calcium intake: 800 mg)

Characteristics		Men	Women
Age, years		−0.11 (−0.12, −0.092)	−0.033 (−0.043, −0.022)
Group			
	1 (n = 208/741)		
	2 (n = 4,594/7,003)	1.73 (0.91, 2.54)	0.68 (0.15, 1.22)
	3 (n = 37/61)	1.51 (−0.44, 3.46)	0.75 (−0.77, 2.27)
	4 (n = 1,017/783)	2.51 (1.59, 3.43)	1.12 (0.48, 1.75)
Body mass index, kg/m^2			
	< 25		
	≥ 25	6.82 (6.45, 7.19)	5.14 (4.85, 5.43)
Nutritional intake			
	Total energy intake, kcal/day	0.002 (0.001, 0.002)	0.0009 (0.0007, 0.001)
	Water intake/body weight, g/kg/day	−0.12 (−0.14, −0.09)	−0.073 (−0.089, −0.056)
Smoking			
	None		
	Past	0.41 (−0.088, 0.91)	0.57 (−0.14, 1.27)
	Current	0.29 (−0.15, 0.73)	−0.35 (−0.83, 0.14)
Alcohol drinking			
	<1 time/month		
	≥1 time/month	0.39 (0.016, 0.76)	0.41 (0.19, 0.62)
Physical activity, MET-min/week		0.0001 (0.00007, 0.0002)	0.0002 (0.0001, 0.0002)
Education			
	≤Elementary school		
	Middle school	1.00 (0.36, 1.64)	0.94 (0.53, 1.34)
	High school	1.43 (0.86, 2.01)	1.29 (0.88, 1.69)
	≥College	1.61 (1.02, 2.21)	1.05 (0.59, 1.50)
Average monthly household income, 10000 KRW		0.002 (0.001, 0.003)	0.0005 (0.00005, 0.001)
Occupation			
	No		
	Yes	0.53 (0.12, 0.93)	0.09 (−0.12, 0.31)
Survey year			
	2008		
	2009	−0.64 (−1.20, −0.072)	−0.022 (−0.38, 0.338)
	2010	−1.41 (−2.02, −0.80)	−0.84 (−1.25, −0.43)
	2011	−0.18 (−0.95, 0.59)	−0.13 (−0.61, 0.34)

MET, Metabolic Equivalent Task; KRW, Korea Republic Won

Data are presented as beta coefficient (95% confidence interval).

Journal of
Clinical Medicine

Group 1: 25-hydroxy vitamin D < 10 ng/mL and calcium intake < 800 mg

Group 2: 25-hydroxy vitamin D ≥ 10 ng/mL and calcium intake < 800 mg

Group 3: 25-hydroxy vitamin D < 10 ng/mL and calcium intake ≥ 800 mg

Group 4: 25-hydroxy vitamin D ≥ 10 ng/mL and calcium intake ≥ 800 mg

[a]Multivariable model adjusted for age, body mass index status, total energy intake, water intake per body weight, smoking, alcohol drinking, physical activity, education, income, occupation, and survey year.

Journal of
Clinical Medicine

Table S4. Linear regression analysis of changes in whole body total fat−free mass by group (serum vitamin D level cutoff: 20 ng/mL, recommended daily calcium intake: 800 mg)

**Characteristics**		Men	Women
Age, years		-0.11 (-0.13, -0.094)	-0.032 (-0.043, -0.022)
Group			
	4451 (n = 2,630/5,497)		
	2 (n = 2,172/2,247)	0.44 (0.028, 0.84)	0.14 (−0.11, 0.40)
	3 (n = 576/588)	0.53 (−0.067, 1.13)	0.37 (−0.05, 0.80)
	4 (n = 478/256)	1.68 (0.94, 2.42)	0.82 (0.17, 1.47)
Body mass index, kg/m^2			
	< 25		
	≥ 25	6.84 (6.47, 7.20)	5.14 (4.86, 5.43)
Nutritional intake			
	Total energy intake, kcal/day	0.002 (0.001, 0.002)	0.0009 (0.0007, 0.001)
	Water intake/body weight, g/kg/day	−0.12 (−0.14, −0.10)	−0.073 (−0.089, −0.056)
Smoking			
	None		
	Past	0.41 (−0.092, 0.91)	0.57 (−0.14, 1.27)
	Current	0.27 (−0.17, 0.71)	−0.35 (−0.83, 0.13)
Alcohol drinking			
	<1 time/month		
	≥1 time/month	0.38 (-0.001, 0.75)	0.43 (0.21, 0.64)
Physical activity, MET-min/week		0.0001 (0.00006, 0.0002)	0.0002 (0.0001, 0.0002)
Education			
	≤Elementary school		
	Middle school	1.05 (0.41, 1.68)	0.94 (0.54, 1.35)
	High school	1.47 (0.89, 2.05)	1.29 (0.88, 1.70)
	≥College	1.68 (1.09, 2.27)	1.05 (0.60, 1.50)
Average monthly household income, 10000 KRW		0.002 (0.001, 0.003)	0.0005 (0.00005, 0.001)
Occupation			
	No		
	Yes	0.55 (0.15, 0.96)	0.10 (−0.11, 0.32)
Survey year			
	2008		
	2009	−0.53 (−1.10, 0.037)	−0.018 (−0.39, 0.351)
	2010	−1.33 (−1.94, −0.71)	−0.84 (−1.26, −0.43)
	2011	−0.04 (−0.82, 0.74)	−0.15 (−0.62, 0.33)

MET, Metabolic Equivalent Task; KRW, Korea Republic Won

Data are presented as beta coefficient (95% confidence interval).

Journal of
Clinical Medicine

Group 1: 25-hydroxy vitamin D < 20 ng/mL and calcium intake < 800 mg

Group 2: 25-hydroxy vitamin D ≥ 20 ng/mL and calcium intake < 800 mg

Group 3: 25-hydroxy vitamin D < 20 ng/mL and calcium intake ≥ 800 mg

Group 4: 25-hydroxy vitamin D ≥ 20 ng/mL and calcium intake ≥ 800 mg

[a]Multivariable model adjusted for age, body mass index status, total energy intake, water intake per body weight, smoking, alcohol drinking, physical activity, education, income, occupation, and survey year.

Table S5. Linear regression analysis of changes in whole body total fat−free mass by group (serum vitamin D level cutoff: 30 ng/mL, recommended daily calcium intake: 800 mg)

**Characteristics**		Men	Women
Age, years		-0.11 (−0.12, −0.092)	−0.032 (−0.043, −0.021)
Group			
	1 (n = 4,326/7,346)		
	2 (n = 476/398)	0.14 (−0.44, 0.71)	0.18 (−0.30, 0.66)
	3 (n = 948/804)	0.68 (0.19, 1.17)	0.42 (0.051, 0.80)
	4 (n = 106/40)	2.64 (1.37, 3.90)	1.77 (0.42, 3.12)
Body mass index, kg/m^2			
	< 25		
	≥ 25	6.84 (6.47, 7.21)	5.15 (4.86, 5.44)
Nutritional intake			
	Total energy intake, kcal/day	0.002 (0.001, 0.002)	0.0009 (0.0007, 0.001)
	Water intake/body weight, g/kg/day	−0.12 (−0.14, −0.10)	−0.072 (−0.089, −0.056)
Smoking			
	None		
	Past	0.42 (−0.079, 0.92)	0.57 (−0.13, 1.28)
	Current	0.25 (−0.19, 0.69)	−0.37 (−0.85, 0.12)
Alcohol drinking			
	<1 time/month		
	≥1 time/month	0.42 (0.038, 0.79)	0.43 (0.21, 0.64)
Physical activity, MET-min/week		0.0001 (0.00007, 0.0002)	0.0002 (0.0001, 0.0002)
Education			
	≤Elementary school		
	Middle school	1.03 (0.39, 1.67)	0.93 (0.53, 1.34)
	High school	1.45 (0.86, 2.04)	1.28 (0.88, 1.69)
	≥College	1.63 (1.02, 2.23)	1.04 (0.59, 1.49)
Average monthly household income, 10000 KRW		0.002 (0.001, 0.003)	0.0005 (0.00005, 0.001)
Occupation			
	No		
	Yes	0.57 (0.17, 0.98)	0.10 (−0.12, 0.32)
Survey year			
	2008		
	2009	−0.63 (−1.19, −0.069)	−0.038 (−0.41, 0.330)
	2010	−1.43 (−2.03, −0.82)	−0.86 (−1.28, −0.45)
	2011	−0.23 (−1.00, 0.55)	−0.18 (−0.64, 0.29)

MET, Metabolic Equivalent Task; KRW, Korea Republic Won

Data are presented as beta coefficient (95% confidence interval).

Journal of
Clinical Medicine

Group 1: 25-hydroxy vitamin D < 30 ng/mL and calcium intake < 800 mg

Group 2: 25-hydroxy vitamin D ≥ 30 ng/mL and calcium intake < 800 mg

Group 3: 25-hydroxy vitamin D < 30 ng/mL and calcium intake ≥ 800 mg

Group 4: 25-hydroxy vitamin D ≥ 30 ng/mL and calcium intake ≥ 800 mg

[a]Multivariable model adjusted for age, body mass index status, total energy intake, water intake per body weight, smoking, alcohol drinking, physical activity, education, income, occupation, and survey year.

Journal of Clinical Medicine

Table S6. Linear regression analysis of changes in whole body total fat−free mass by group (serum vitamin D level cutoff: 10 ng/mL, recommended daily calcium intake: 1000 mg)

**Characteristics**		Men	Women
Age, years		−0.11 (−0.12, −0.092)	−0.033 (−0.043, −0.022)
Group			
	1 (n = 222/778)		
	2 (n = 5,122/7,397)	1.62 (0.83, 2.41)	0.70 (0.17, 1.23)
	3 (n = 23/24)	1.11 (−1.31, 3.54)	1.47 (−0.86, 3.79)
	4 (n = 489/389)	3.03 (2.01, 4.04)	0.96 (0.33, 1.58)
Body mass index, kg/m^2			
	< 25		
	≥ 25	6.82 (6.45, 7.19)	5.14 (4.86, 5.43)
Nutritional intake			
	Total energy intake, kcal/day	0.002 (0.001, 0.002)	0.0009 (0.0007, 0.001)
	Water intake/body weight, g/kg/day	−0.12 (−0.14, −0.09)	−0.071 (−0.087, −0.055)
Smoking			
	None		
	Past	0.41 (−0.090, 0.91)	0.57 (−0.14, 1.28)
	Current	0.31 (−0.13, 0.75)	−0.35 (−0.83, 0.14)
Alcohol drinking			
	<1 time/month		
	≥1 time/month	0.39 (0.017, 0.76)	0.40 (0.19, 0.61)
Physical activity, MET-min/week		0.0001 (0.00006, 0.0002)	0.0002 (0.0001, 0.0002)
Education			
	≤Elementary school		
	Middle school	1.01 (0.36, 1.65)	0.95 (0.55, 1.36)
	High school	1.44 (0.87, 2.02)	1.30 (0.89, 1.70)
	≥College	1.64 (1.04, 2.23)	1.05 (0.60, 1.51)
Average monthly household income, 10000 KRW		0.002 (0.001, 0.003)	0.0005 (0.00004, 0.001)
Occupation			
	No		
	Yes	0.54 (0.14, 0.95)	0.10 (−0.12, 0.31)
Survey year			
	2008		
	2009	−0.63 (−1.20, −0.067)	−0.018 (−0.38, 0.342)
	2010	−1.40 (−2.02, −0.79)	−0.83 (−1.24, −0.42)
	2011	−0.18 (−0.95, 0.58)	−0.13 (−0.60, 0.34)

MET, Metabolic Equivalent Task; KRW, Korea Republic Won

Data are presented as beta coefficient (95% confidence interval).

Journal of
Clinical Medicine

Group 1: 25-hydroxy vitamin D < 10 ng/mL and calcium intake < 1000 mg

Group 2: 25-hydroxy vitamin D ≥ 10 ng/mL and calcium intake < 1000 mg

Group 3: 25-hydroxy vitamin D < 10 ng/mL and calcium intake ≥ 1000 mg

Group 4: 25-hydroxy vitamin D ≥ 10 ng/mL and calcium intake ≥ 1000 mg

[a]Multivariable model adjusted for age, body mass index status, total energy intake, water intake per body weight, smoking, alcohol drinking, physical activity, education, income, occupation, and survey year.

Journal of
Clinical Medicine

Table S7. Linear regression analysis of changes in whole body total fat−free mass by group (serum vitamin D level cutoff: 20 ng/mL, recommended daily calcium intake: 1000 mg)

**Characteristics**		Men	Women
Age, years		-0.11 (-0.13, -0.094)	-0.032 (-0.043, -0.021)
Group			
	1 (n = 2,936/5,801)		
	2 (n = 2,408/2,374)	0.45 (0.060, 0.84)	0.17 (−0.08, 0.42)
	3 (n = 270/284)	0.83 (0.041, 1.62)	0.31 (−0.19, 0.80)
	4 (n = 242/129)	2.50 (1.57, 3.44)	0.60 (−0.18, 1.38)
Body mass index, kg/m^2			
	< 25		
	≥ 25	6.83 (6.46, 7.19)	5.15 (4.86, 5.43)
Nutritional intake			
	Total energy intake, kcal/day	0.002 (0.001, 0.002)	0.0009 (0.0007, 0.001)
	Water intake/body weight, g/kg/day	−0.12 (−0.14, −0.10)	−0.071 (−0.087, −0.055)
Smoking			
	None		
	Past	0.40 (−0.095, 0.90)	0.57 (−0.13, 1.28)
	Current	0.27 (−0.17, 0.71)	−0.35 (−0.83, 0.13)
Alcohol drinking			
	<1 time/month		
	≥1 time/month	0.38 (0.001, 0.75)	0.42 (0.20, 0.63)
Physical activity, MET-min/week		0.0001 (0.00006, 0.0002)	0.0002 (0.0001, 0.0002)
Education			
	≤Elementary school		
	Middle school	1.06 (0.43, 1.70)	0.95 (0.55, 1.36)
	High school	1.49 (0.91, 2.07)	1.30 (0.89, 1.71)
	≥College	1.71 (1.12, 2.29)	1.06 (0.60, 1.51)
Average monthly household income, 10000 KRW		0.002 (0.001, 0.003)	0.0005 (0.00004, 0.001)
Occupation			
	No		
	Yes	0.56 (0.16, 0.96)	0.10 (−0.12, 0.32)
Survey year			
	2008		
	2009	−0.53 (−1.10, 0.037)	-0.014 (−0.38, 0.356)
	2010	−1.33 (−1.94, −0.71)	−0.83 (−1.25, −0.42)
	2011	−0.06 (−0.84, 0.72)	−0.13 (−0.61, 0.34)

MET, Metabolic Equivalent Task; KRW, Korea Republic Won

Data are presented as beta coefficient (95% confidence interval).

Group 1: 25-hydroxy vitamin D < 20 ng/mL and calcium intake < 1000 mg

Group 2: 25-hydroxy vitamin D ≥ 20 ng/mL and calcium intake < 1000 mg

Group 3: 25-hydroxy vitamin D < 20 ng/mL and calcium intake ≥ 1000 mg

Group 4: 25-hydroxy vitamin D ≥ 20 ng/mL and calcium intake ≥ 1000 mg

[a]Multivariable model adjusted for age, body mass index status, total energy intake, water intake per body weight, smoking, alcohol drinking, physical activity, education, income, occupation, and survey year.

Journal of Clinical Medicine

Table S8. Linear regression analysis of changes in whole body total fat−free mass by group (serum vitamin D level cutoff: 30 ng/mL, recommended daily calcium intake: 1000 mg)

Characteristics		Men	Women
Age, years		−0.11 (−0.12, −0.091)	−0.032 (−0.043, −0.021)
Group			
	1 (n = 4,820/7,760)		
	2 (n = 524/415)	0.44 (−0.15, 1.03)	0.24 (−0.23, 0.70)
	3 (n = 454/390)	1.33 (0.69, 1.98)	0.31 (−0.12, 0.74)
	4 (n = 58/23)	1.88 (0.48, 3.28)	1.27 (−0.71, 3.25)
Body mass index, kg/m^2			
	< 25		
	≥ 25	6.84 (6.47, 7.21)	5.15 (4.87, 5.44)
Nutritional intake			
	Total energy intake, kcal/day	0.002 (0.001, 0.002)	0.0009 (0.0007, 0.001)
	Water intake/body weight, g/kg/day	−0.12 (−0.14, −0.09)	−0.070 (−0.086, −0.054)
Smoking			
	None		
	Past	0.41 (−0.089, 0.91)	0.58 (−0.13, 1.28)
	Current	0.25 (−0.18, 0.69)	−0.36 (−0.84, 0.12)
Alcohol drinking			
	<1 time/month		
	≥1 time/month	0.40 (0.030, 0.78)	0.41 (0.20, 0.63)
Physical activity, MET-min/week		0.0001 (0.00006, 0.0002)	0.0002 (0.0001, 0.0002)
Education			
	≤Elementary school		
	Middle school	1.03 (0.38, 1.67)	0.94 (0.54, 1.35)
	High school	1.46 (0.87, 2.05)	1.29 (0.88, 1.70)
	≥College	1.65 (1.05, 2.25)	1.05 (0.59, 1.50)
Average monthly household income, 10000 KRW		0.002 (0.001, 0.003)	0.0005 (0.00004, 0.001)
Occupation			
	No		
	Yes	0.58 (0.18, 0.99)	0.10 (−0.12, 0.32)
Survey year			
	2008		
	2009	−0.62 (−1.18, −0.053)	−0.035 (−0.40, 0.333)
	2010	−1.41 (−2.02, −0.80)	−0.86 (−1.27, −0.44)
	2011	−0.22 (−1.00, 0.55)	−0.17 (−0.63, 0.30)

MET, Metabolic Equivalent Task; KRW, Korea Republic Won

Data are presented as beta coefficient (95% confidence interval).

Group 1: 25-hydroxy vitamin D < 30 ng/mL and calcium intake < 1000 mg

Group 2: 25-hydroxy vitamin D ≥ 30 ng/mL and calcium intake < 1000 mg

Group 3: 25-hydroxy vitamin D < 30 ng/mL and calcium intake ≥ 1000 mg

Group 4: 25-hydroxy vitamin D ≥ 30 ng/mL and calcium intake ≥ 1000 mg

[a]Multivariable model adjusted for age, body mass index status, total energy intake, water intake per body weight, smoking, alcohol drinking, physical activity, education, income, occupation, and survey year.

Journal of
Clinical Medicine

MDPI

Table S9. Linear regression analysis of changes in whole body total fat−free mass according to serum vitamin D level and daily calcium intake

**Characteristics**		Men	Women
Age, years		−0.11 (−0.13, −0.10)	−0.034 (−0.044, −0.023)
25-hydroxy vitamin D, ng/mL		0.061 (0.035, 0.086)	0.023 (0.003, 0.043)
Calcium intake, g		1.13 (0.59, 1.68)	0.62 (0.067, 1.16)
Body mass index, kg/m^2			
	< 25		
	≥ 25	6.83 (6.46, 7.20)	5.14 (4.85, 5.42)
Nutritional intake			
	Total energy intake, kcal/day	0.001 (0.001, 0.002)	0.0009 (0.0006, 0.001)
	Water intake/body weight, g/kg/day	−0.12 (−0.15, −0.10)	−0.076 (−0.093, −0.058)
Smoking			
	None		
	Past	0.39 (−0.11, 0.89)	0.57 (−0.14, 1.27)
	Current	0.29 (−0.15, 0.74)	−0.33 (−0.81, 0.16)
Alcohol drinking			
	<1 time/month		
	≥1 time/month	0.37 (−0.006, 0.75)	0.42 (0.20, 0.63)
Physical activity, MET-min/week		0.0001 (0.00005, 0.0002)	0.0002 (0.0001, 0.0002)
Education			
	≤Elementary school		
	Middle school	1.04 (0.39, 1.68)	0.92 (0.52, 1.33)
	High school	1.49 (0.91, 2.07)	1.28 (0.87, 1.69)
	≥College	1.70 (1.11, 2.29)	1.04 (0.58, 1.49)
Average monthly household income, 10000 KRW		0.002 (0.001, 0.003)	0.0005 (0.00005, 0.001)
Occupation			
	No		
	Yes	0.49 (0.080, 0.89)	0.11 (−0.11, 0.32)
Survey year			
	2008		
	2009	−0.43 (−0.99, 0.132)	0.024 (−0.34, 0.39)
	2010	−1.21 (−1.82, −0.59)	−0.80 (−1.22, −0.39)
	2011	0.12 (−0.67, 0.90)	−0.07 (−0.55, 0.40)

MET, Metabolic Equivalent Task; KRW, Korea Republic Won

Data are presented as beta coefficient (95% confidence interval).

[a]Multivariable model adjusted for age, body mass index status, total energy intake, water intake per body weight, smoking, alcohol drinking, physical activity, education, income, occupation, and survey year.
